# Genetic and Epigenetic Mechanisms in Serrated Adenocarcinomas and Classical Colorectal Carcinomas: An In Silico Study

**DOI:** 10.3390/cimb48020179

**Published:** 2026-02-04

**Authors:** Zeynep Sagnak Yilmaz, Sibel Demir Kececi, Ozgul Sagol, Sulen Sarioglu

**Affiliations:** 1 Department of Pathology, Faculty of Medicine, Karadeniz Technical University, 61080 Trabzon, Türkiye; 2 Department of Molecular Pathology, Graduate School of Health Sciences, Dokuz Eylul University, 35220 Izmir, Türkiye; sibeldemir2840@gmail.com (S.D.K.); ozgul.sagol@deu.edu.tr (O.S.); 3 Department of Pathology, Manisa City Hospital, 45040 Manisa, Türkiye; 4 Department of Pathology, Faculty of Medicine, Dokuz Eylul University, 35220 Izmir, Türkiye; 5 Department of Pathology, Faculty of Medicine, Izmir University of Economics, 35330 Izmir, Türkiye; sulensari@gmail.com; 6 Department of Pathology, Memorial Health Group, 34676 Istanbul, Türkiye

**Keywords:** colorectal carcinoma, serrated adenocarcinoma, in silico, somatic mutation, epigenetic

## Abstract

Serrated adenocarcinoma (SAC) represents a molecularly heterogeneous subtype of colorectal carcinoma (CRC) linked to the serrated pathway. It is aimed to clarify the molecular mechanisms underlying SAC development. Digital slides from The Cancer Genome Atlas (TCGA) colorectal adenocarcinoma Firehose Legacy dataset (632 cases) were reviewed, and cases were classified as SAC, partial-SAC, or classical CRC. Genomic alterations, mRNA expression, and DNA hypermethylation were compared using cBioPortal. Enrichment analyses were performed via WebGestalt, and protein–protein interaction (PPI) networks with hub genes were identified using STRING and Cytoscape. Statistical significance was defined as *p* < 0.05 and *q* < 0.05. The results revealed that the groups showed significant differences in the expression of 327 genomic alterations, 20 mRNAs, and 21 methylated genes (*p* < 0.0001, *q* < 0.0001). Hub genes were *PSMC1*, *FLT3LG*, *SNW1*, *H3C2*, *H1-2*, *H2BC14*, *H1-5*, *RPS16*, *SUPT5H*, and *MYOD1*. The pathways associated with differently expressed genes were the following: cell structure and morphology (phagocytic vesicle, microvillus, endocytosis, and immobile cilium), protein kinase activity (particularly MAPK), and immunological mechanisms. The hub genes act as molecular bridges connecting the observed genomic and epigenetic variations, particularly driving chromatin-related regulation and MAPK signaling pathways. In particular, *PSMC1*, *SNW1*, *H3C2*, *H1-2*, and *H2BC14* genes offer promising molecular targets for future therapeutic approaches in SACs.

## 1. Introduction

Colorectal carcinoma (CRC) is the third most common type of cancer worldwide and the second most common cause of cancer-related death. According to recent epidemiological data, CRC continues to represent a significant global health challenge, as it is the third most frequently diagnosed malignancy and the second leading cause of cancer-associated mortality worldwide. In 2022, approximately 1.9 million new cases were diagnosed, resulting in an estimated 904,000 deaths globally [1]. Serrated adenocarcinoma (SAC) was first described by Jass and Smith in 1992 and has been included as a subtype of CRCs since 2010 under the World Health Organization (WHO) classification [2]. SAC is a serrated pathway-associated neoplasm of CRCs and is a molecularly heterogeneous subtype. It is detected in 5.8–12% of CRCs and is characterized by epithelial serration, eosinophilic cytoplasm, vesicular nucleus, papillary rod structures, and clusters [3,4,5]. Although SACs can develop from traditional serrated adenoma (TSA) or sessile serrated lesion (SSL), molecular mechanisms such as *KRAS* or *BRAF* mutation, microsatellite instability (MSI), and hypermethylation of CpG islands are also known to be effective in this pathway [6]. Additionally, SAC showed differences in the expression of proteins involved in morphogenesis, hypoxia, cytoskeleton, and vesicle transport compared to classical CRC. It has been emphasized that the mechanism responsible for serration is apoptosis inhibition, and genes related to cytoskeleton-related function and cell components are effective in serrated morphology [5,7,8]. Although the first studies on SACs were mostly associated with a poor prognosis, some studies had shown no difference in mortality between classical CRCs and SACs [9,10,11]. The molecular differences between SAC and classical CRC have not yet been fully elucidated. Considering that carcinomas developing from TSA or SSL are classical CRC rather than SAC, it is important to understand the molecular interactions involved in this complex mechanism [3]. Previous computational studies have demonstrated that integrating large-scale genomic datasets, such as The Cancer Genome Atlas Program (TCGA), with network-based bioinformatics tools is a powerful strategy for identifying novel biomarkers and therapeutic targets in various malignancies [12,13]. Previous in silico studies have also investigated cancer-related pathways and hub genes in CRCs [14]. While in silico approaches have been successfully applied to CRCs, there is a notable scarcity of studies specifically focused on the distinct molecular architecture of SAC [15]. The aim of this study is to elucidate the molecular mechanisms involved in the development of SACs and to reveal the genetic and epigenetic alterations in the pathogenesis of SACs that differ from classical CRCs through in silico analysis.

## 2. Materials and Methods

### 2.1. Data Set

The molecular and clinical data in the study were obtained from the public bioinformatics database cBioPortal for Cancer Genomics (cBioPortal) website (https://www.cbioportal.org, accessed on 11 January 2026). The Firehose Legacy dataset from TGCA was used. This dataset includes 640 cases of colorectal adenocarcinoma. The Center for Cancer Genomics data was accessed from TCGA through the National Cancer Institute (NCI) website (https://www.cancer.gov/ccg/research/genome-sequencing/tcga, accessed on 11 January 2026). TCGA serial numbers of the cases in the Colorectal adenocarcinoma, TCGA, Firehose Legacy dataset were uploaded, and the digital slides of the cases were accessed. Since 4 cases had the same serial number and the digital slides of 4 cases were not uploaded, 632 cases were included in the study.

### 2.2. Histopathological Examination

**Figure 1:**
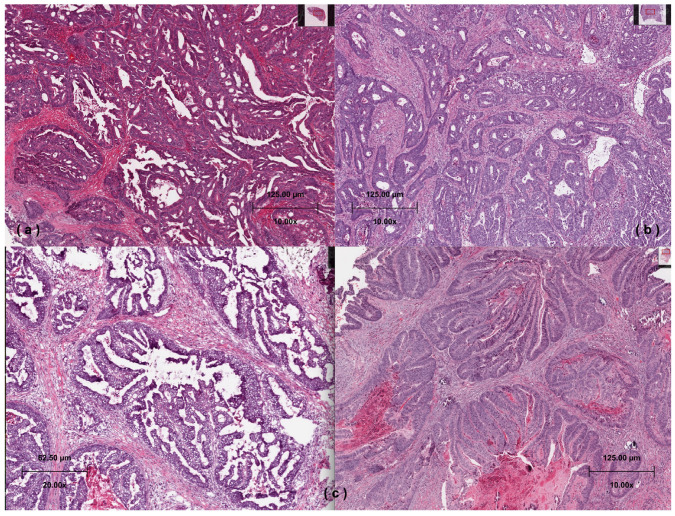
(**a**): Serrated adenocarcinoma composed of epithelial serrations, eosinophilic cytoplasm, and papillary rods (hematoxylin and eosin) (TCGA-A6-6650). (**b**) Classical CRC—case without serrated features (hematoxylin and eosin) (TCGA-D5-5540). (**c**) Partial-SAC—case with less than 50% serrated morphology/the tumor showing serrated features on the left and not showing serrated features on the right (hematoxylin and eosin) (TCGA-A6-5656).

Digital slides of 632 cases were histopathologically analyzed for SAC morphology. Cases with the morphological characteristics of SAC are defined using Makinen’s [16] criteria; such as epithelial serrations, eosinophilic or clear cytoplasm, abundant cytoplasm, vesicular nuclei, distinct nucleoli, and preserved polarity, necrosis in less than 10% of the total area or absent of necrosis and the presence of cell balls and papillary rods within mucinous areas (Figure 1a/TCGA-A6-6650, https://portal.gdc.cancer.gov/image-viewer/MultipleImageViewerPage?caseId=6cd8d2dd-5545-43b0-b796-ae0506d62eb8, accessed on 11 January 2026). Serrated-like structures resulting from tumor cell necrosis were excluded [17]. As in previous studies, cases with more than 50% serrated morphology were considered as SAC [18]. Whole-slide digital images were scanned at 20× magnification, and necrosis was quantified across all tumor-containing regions to ensure the <10% cut off was strictly applied. Cases without serrated features were grouped as classical CRC (Figure 1b/TCGA-D5-5540, https://portal.gdc.cancer.gov/image-viewer/MultipleImageViewerPage?caseId=31d960d7-8f5b-4d22-bf5e-fae0849c4efd, accessed on 11 January 2026). Cases with less than 50% serrated morphology were grouped as CRC with partial-SAC morphology (partial-SAC) (Figure 1c/TCGA-A6-5656, https://portal.gdc.cancer.gov/image-viewer/MultipleImageViewerPage?caseId=eb4e4e09-98b3-4e85-8dd2-75676ff2af14, accessed on 11 January 2026).

The histology was confirmed by two independent pathologists (S.Y.Z and S.S) from the haematoxylin and eosin-stained digital slides. Based on the examination of the digital slides, we classified the cases into three histopathologic subgroups: SAC, partial-SAC, and classical CRC.

### 2.3. Identification of Differentially Expressed Genes

Genomic differences between the three groups (SAC, partial-SAC, and classical CRC) and between the two groups (SAC and classical CRC) were analyzed in terms of ‘genomic alterations’, ‘mRNA expression’, and ‘DNA methylation’ using cBioPortal comparison analysis. The platform provides both *p*-values and multiple-testing adjusted *q*-values. *q*-values were calculated using the Benjamini–Hochberg false discovery rate (FDR) correction method implemented in the cBioPortal differential expression algorithm. Genes with *p* < 0.05 and *q* < 0.05 were considered statistically significant. Differentially expressed genes (DEGs) were identified between the groups.

### 2.4. Microsatellite İnstability

We obtained MSI information for approximately half of our cases (*n* = 275) from the Colorectal Adenocarcinoma TCGA, Nature 2012 dataset. SAC, partial-SAC, and classical CRC cases were grouped as microsatellite stable (MSS), microsatellite instability-Low (MSI-L), and microsatellite instability-High (MSI-H) via cBioPortal.

### 2.5. Survival Analysis and Clinicopathological Features

Survival analyses were performed to compare disease-free survival (DFS) and overall survival (OS) among classical CRC, partial-SAC, and SAC groups through cBioPortal. Kaplan–Meier curves were generated for each subgroup, and differences were evaluated using the log-rank test. Median survival times were calculated by the cBioPortal survival module. Statistical significance was accepted as *p* < 0.05.

Clinicopathological features of the study cohorts, including the American Joint Committee on Cancer (AJCC) pathological T, N, and M stages, as well as the clinical stage, were retrieved from the cBioPortal. These categorical variables were compared across the SAC, partial-SAC, and classical CRC groups using the chi-square test or Fisher’s exact test to assess the homogeneity of the cohorts at the time of diagnosis. Statistical significance was accepted as *p* < 0.05.

### 2.6. Pathway Analysis

Pathways associated with DEGs were accessed through the WEB-based Gene SeT Analysis Toolkit (WebGestalt) (https://www.webgestalt.org, accessed on 11 January 2026), a public bioinformatics database [19]. Over-representation analysis (ORA) was used for enrichment analysis. Biological Process (BP), Molecular Function (MF), and Cellular Component (CC) pathways were accessed through Gene Ontology (GO). Kyoto Encyclopedia of Genes and Genomes (KEGG) pathway analysis was also performed. ‘Genome’ was selected as the reference set. Enrichment analyses were conducted for the following: genomic alterations showing significant *q*-values in cBioPortal, differentially expressed mRNAs (upregulated and downregulated subsets), and differentially methylated genes (hypermethylated and hypomethylated subsets). Gene sets exhibiting *p* < 0.05 and FDR < 0.05 were identified as significantly enriched. Results were evaluated based on both the enrichment ratio and the FDR. However, to ensure a comprehensive biological interpretation, certain pathways demonstrating a high enrichment ratio were also included for qualitative discussion as notable biological propensities, even if they exceeded the formal statistical significance threshold (FDR > 0.05).

### 2.7. Detection of Protein–Protein Interactions and Hub Genes

The Search Tool for the Retrieval of Interacting Genes/Proteins (STRING) online tool (https://string-db.org/, version 12.0, accessed on 11 January 2026) was used to detect protein–protein interaction (PPI) in DEGs [20]. PPIs with a combined score above 0.4 were significant. Significant PPIs were submitted to cytoHubba, plug-in Cytoscape (https://cytoscape.org, version 3.8.2, accessed on 11 January 2026), and the top 10 most interacting hub genes were identified. In the PPI network, genes that have many connections (interactions) with other genes and control/regulate the network structure are hub genes [21,22]. The prognostic significance of the identified hub genes was evaluated via the cBioPortal. OS and DFS were compared between patients with high and low mRNA expression of the hub genes. The statistical significance of differences between the groups was determined using the log-rank test. A *p*-value of <0.05 was considered statistically significant, while a *p*-value of <0.25 was considered to indicate a potential clinical trend, as suggested for prioritized biomarker identification in exploratory cohorts [23].

**Figure 2:**
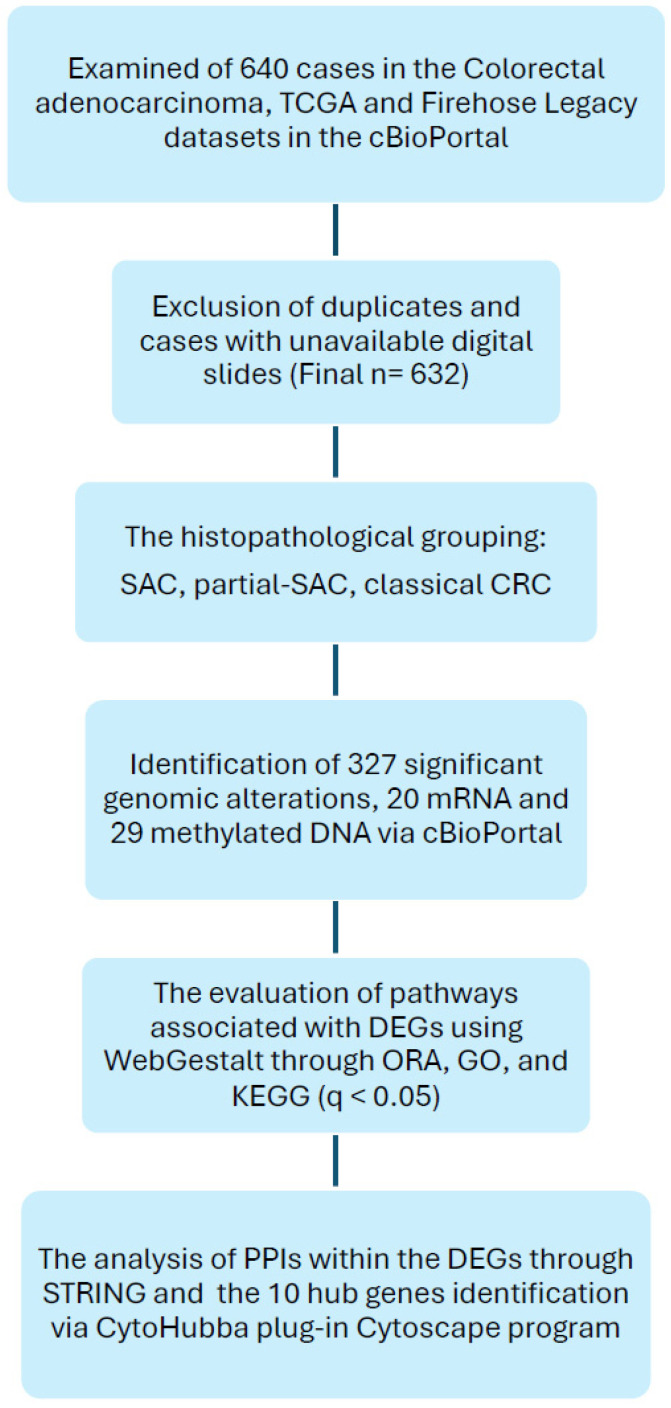
Flowchart of cases, evaluated parameters, and databases.

The flow chart for the selection of cases, the parameters evaluated, and the databases used are shown in Figure 2.

## 3. Results

A total of 632 cases were analyzed using digital slides from the TCGA database. In total, 546 cases (86.4%) were classical CRC, 59 cases (9.3%) had SAC morphology, and 27 cases (4.3%) had partial-SAC morphology. Cases classified as SAC and partial-SAC are presented in Supplementary Table S1 with the TCGA patient number.

### 3.1. Differentially Expressed/Methylated Genes

#### 3.1.1. Genomic Alterations

Significant genomic alterations were detected in 327 genes between SAC, partial-SAC, and classical CRC groups (Supplementary Table S2). In total, 161 genes were enriched in SACs, and 166 genes were enriched in partial-SACs. The 10 most significant genes were as follows: genes enriched in SACs—*COX6A2* (*q =* 1.157 × 10^−3^), *CHGA* (*q =* 2.085 × 10^−3^), *COX8C* (*q =* 2.085 × 10^−3^), *AHSP* (*q =* 5.072 × 10^−3^), and *CLUHP3* (*q =* 5.072 × 10^−3^); genes enriched in partial-SACs—*CAPRIN1* (*q* = 1.298 × 10^−3^), *DENND5A* (*q* = 1.298 × 10^−3^), *RPS16* (*q* = 1.298 × 10^−3^), *ACAD10* (*q* = 2.085 × 10^−3^), and *ZNF146* (*q* = 2.085 × 10^−3^).

#### 3.1.2. mRNA

**Figure 3:**
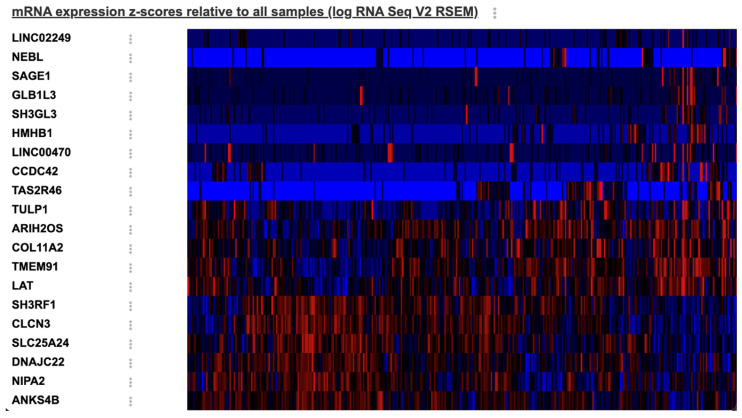
The heat map for differentially expressed mRNAs between SAC and classical CRC groups (red—higher expression, blue—lower expression).

Twenty DEGs showed a significant difference between SAC and classical CRC. The heat map is shown in Figure 3. The 10 most significant genes were as follows: *SH3RF1* (*q* = 9.498 × 10^−3^) and *CLCN3* (*q* = 9.498 × 10^−3^) genes had higher expression in SACs, and *HMHB1* (*q* = 6.718 × 10^−5^), *SH3GL3* (*q* = 1.790 × 10^−4^), *LINC02249* (*q* = 2.478 × 10^−3^), *GLB1L3* (*q* = 3.530 × 10^−3^), *TAS2R46* (*q* = 9.498 × 10^−3^), *NEBL* (*q* = 9.498 × 10^−3^), *LINC00470* (*q* = 0.0143), and *CCDC42* (*q* = 0.0153) genes had higher expression in classical CRCs.

#### 3.1.3. DNA Methylation

**Figure 4:**
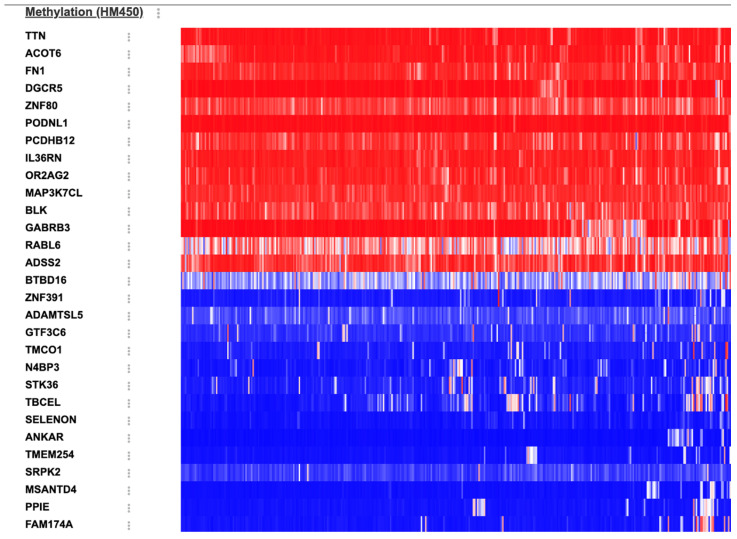
The heat map for differentially methylated genes between SAC and classical CRC groups (red—hypermethylation, blue—hypomethylation).

Twenty-nine genes exhibited significant hypermethylation when analyzed in terms of methylation status between SAC and classical CRC cases. The volcano plot and heat map are shown in Figure 4. The top 10 genes with the highest significance were as follows: *ZNF80* (*q =* 1.256 × 10^−6^), *TTN* (*q =* 5.982 × 10^−5^), *OR2AG2* (*q =* 3.779 × 10^−3^), *DGCR5* (*q =* 5.911 × 10^−3^), and *MAP3K7CL* (*q =* 8.716 × 10^−3^) genes exhibited higher frequency of hypermethylation in SACs, and *TBCEL* (*q =* 7.016 × 10^−4^), *BTBD16* (*q =* 3.779 × 10^−3^), *GTF3C6* (*q =* 4.621 × 10^−3^), *TMCO1* (*q =* 4.621 × 10^−3^), and *PPIE* (*q =* 0.0105) genes exhibited higher frequency of hypermethylation in classical CRCs.

### 3.2. Microsatellite Instability

MSI information of 275 cases was obtained and examined. Of the 25 SAC cases, 19 cases (76%) were MSS, 3 cases (12%) were MSI-L, and 3 cases (12%) were MSI-H. Of the 235 classical CRC cases, 165 cases were MSS (70.2%), 37 cases (15.8%) were MSI-L, and 33 cases (14%) were MSI-H. Of the 15 partial-SAC cases, 9 cases (60%) were MSS, 4 cases (26.7%) were MSI-L, and 2 cases (13.3%) were MSI-H. No statistically significant differences were found in MSI among the SAC, partial-SAC, and classic CRC groups (*p*> 0.05 and *q* > 0.05).

### 3.3. Survival and Clinicopathological Features

The median DFS was 108.94 months for classical CRC, 71.48 months for SAC, and 55.12 months for partial-SAC. Although the median DFS appeared shorter in serrated subgroups, no statistically significant difference was observed among the three groups (*p* = 0.944). The median OS was 81.31 months in the classical CRC group and 99.93 months in the partial-SAC group. Due to the limited number of cases and relatively short follow-up in the SAC group, the median OS could not be calculated. There was no statistically significant difference in OS between the three groups (*p* = 0.965). SAC and partial-SAC groups exhibited survival patterns similar to classical CRC throughout the follow-up period; however, fluctuations were observed in the serrated groups due to the lower number of patients. The serrated components did not appear to result in a survival disadvantage in the present dataset.

**Figure 5:**
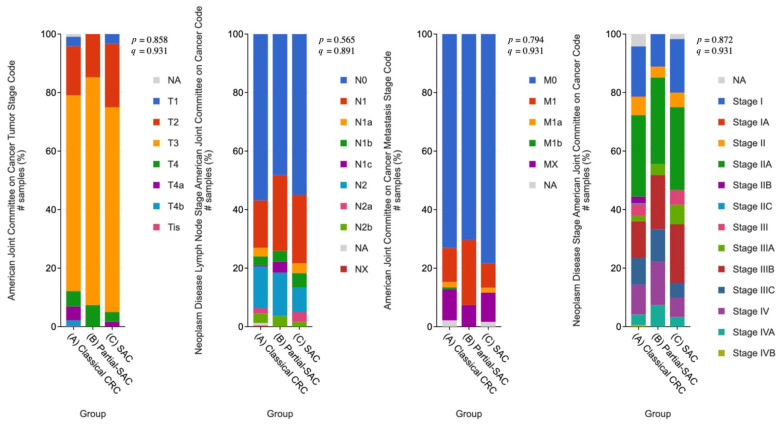
Distribution of clinical stages across SAC, partial-SAC, and classical CRC groups. The bar charts illustrate the percentage distribution of pathological T, N, M stage, and clinical stage among the three groups. #: number of samples.

Clinicopathological characteristics, including T, N, and M stages, as well as the clinical stage, showed no statistically significant differences across the SAC, partial-SAC, and classical CRC groups (*p* > 0.05) for all parameters (Figure 5).

### 3.4. Gene Ontology Enrichment Analysis

#### 3.4.1. Genomic Alterations

The WebGestalt database was used to analyze the pathways associated with DEG genomic alterations detected among SAC, partial-SAC, and classical CRC groups. The most enriched genomic alterations in SACs were examined for BP, MF, and CC pathways through over-representation analysis (GO).

**Figure 6:**
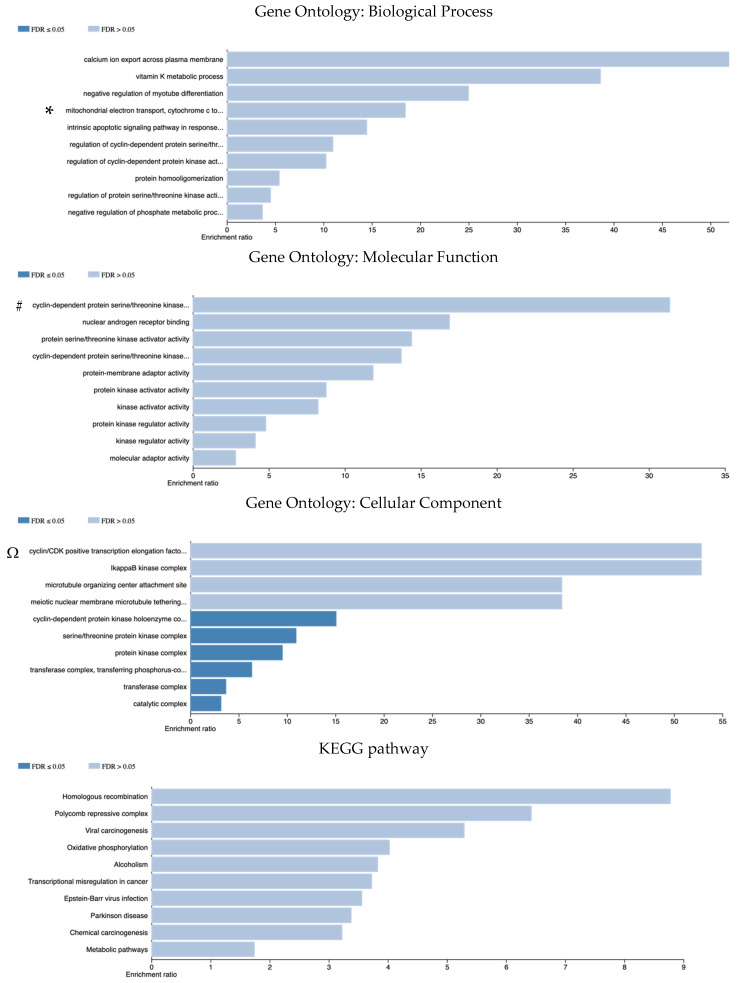
The pathway analysis for DEGs of genomic alterations enriched in SACs. Dark blue bars indicate statistically significant pathways (FDR ≤ 0.05), while light blue bars highlight identified biological propensities with high enrichment ratios, included due to their functional relevance to the SAC molecular landscape. * Mitochondrial electron transport, cytochrome c to oxygen - intrinsic apoptotic signaling pathway in response to DNA damage by p53 class mediator - regulation of cyclin-dependent protein serine/threonine kinase activity - regulation of cyclin-dependent protein kinase activity - regulation of protein serine/threonine kinase activity - negative regulation of phosphate metabolic process. # cyclin-dependent protein serine/threonine kinase regulator activity - cyclin-dependent protein serine/threonine kinase activator activity. Ω cyclin/CDK positive transcription elongation factor complex - meiotic nuclear membrane microtubule tethering complex - cyclin-dependent protein kinase holoenzyme complex - transferase complex, transferring phosphorus-containing groups.

According to GO (BP), genes enriched in SACs were associated with calcium ion export, vitamin K metabolic process, intrinsic apoptotic signaling, and regulation of various protein kinase activities; to GO (MF), genes were associated with protein kinase and kinase activity and regulation; and to GO (CC), genes were related to the cyclin-dependent protein kinase holoenzyme complex, serine/threonine protein kinase complex, protein kinase complex, transferase complex, catalytic complex. The KEGG pathway analysis revealed that these genes were linked to homologous recombination, transcriptional misregulation in cancer, metabolic pathways, and oxidative phosphorylation (Figure 6).

#### 3.4.2. mRNAs

According to GO (BP), the upregulated genes in SACs were associated with negative regulation of cell volume, T-cell proliferation, and mitochondrial ATP transmembrane transport. These genes were related to inorganic phosphate antiporter and transporter activity, MAP-kinase activity, as per GO (MF), and endosome, phagocytic vesicle, and microvillus cell components, as per GO (CC) (Supplementary Figure S1).

In accordance with the GO, BP downregulated genes in SACs were associated with cellular component assembly involved in morphogenesis, endocytosis, and non-motile cilium pathways.

**Figure 7:**
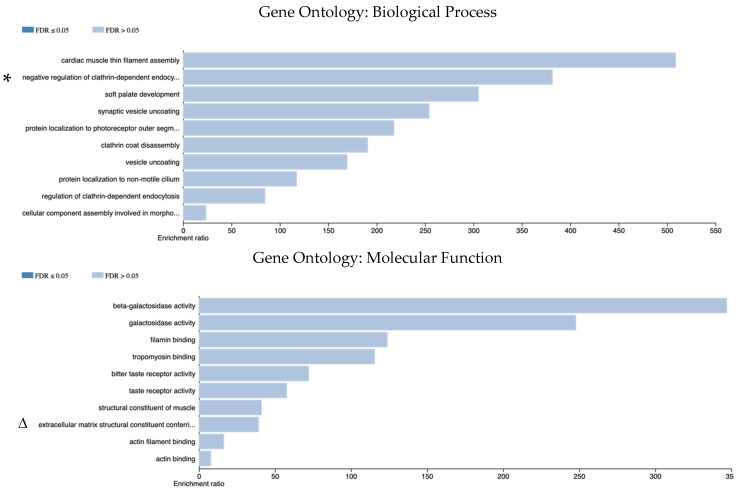

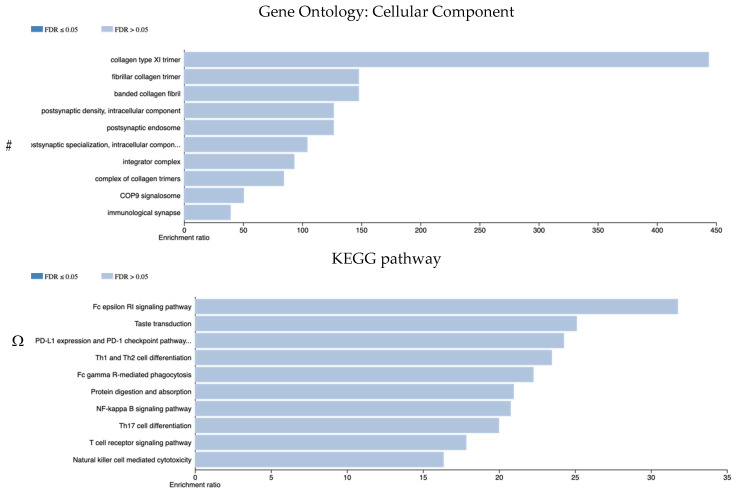
The pathway analysis for DEGs of mRNAs which were downregulated in SACs. The illustrated pathways are identified based on their high enrichment ratios and notable biological propensity within the SAC molecular landscape. * Negative regulation of clathrin-dependent endocytosis - protein localization to photoreceptor outer segment - cellular component assembly involved in morphogenesis. Δ extracellular matrix structural constituent conferring tensile strength. # postsynaptic specialization, intracellular component. Ω PD-L1 expression and PD-1 checkpoint pathway in cancer.

According to GO (MF), functions toward galactosidase activity, actin binding, and taste receptor activity were identified, and via GO (CC), intracellular component, immunological synapse, and postsynaptic specialization pathways were detected. According to the KEGG pathway analysis, the downregulated mRNA DEGs in SACs were linked to immunological pathways, including PD-L1 expression, the PD-1 checkpoint pathway, Th1-2 cell differentiation, the T-cell receptor signaling pathway, and the NF-κB signaling pathway (Figure 7).

#### 3.4.3. DNA Methylation

According to GO (BP), hypermethylated genes in SACs were involved in the negative regulation of monocyte activation, substrate-dependent cell migration, and myosin thick filament assembly. Based on GO (MF), these genes were associated with protein tyrosine kinase activity, interleukin-1 binding, and interleukin-1 receptor binding. Also, according to GO (CC), they were relevant to the GABA receptor complex, the fibrinogen complex, and the condensed nuclear chromosome. KEGG pathway enrichment analysis of the hypermethylated DNA genes revealed associations with alanine, aspartate, and glutamate metabolism, B-cell receptor signaling, ECM-receptor interaction, and GABAergic synapse (Supplementary Figure S2).

DEGs hypomethylated in SACs were associated with the viral process and replication through GO (BP). Based on GO (MF), hypomethylated genes were associated with transcription factor activity, microfibril binding, protein serine kinase activity, poly(A) binding, alpha-tubulin binding, and isomerase activity. According to GO (CC), transcription factor and RNA polymerase-associated complexes, nuclear body and speck, and spliceosomal complex pathways were detected (Supplementary Figure S3).

### 3.5. Hub Genes

**Figure 8:**
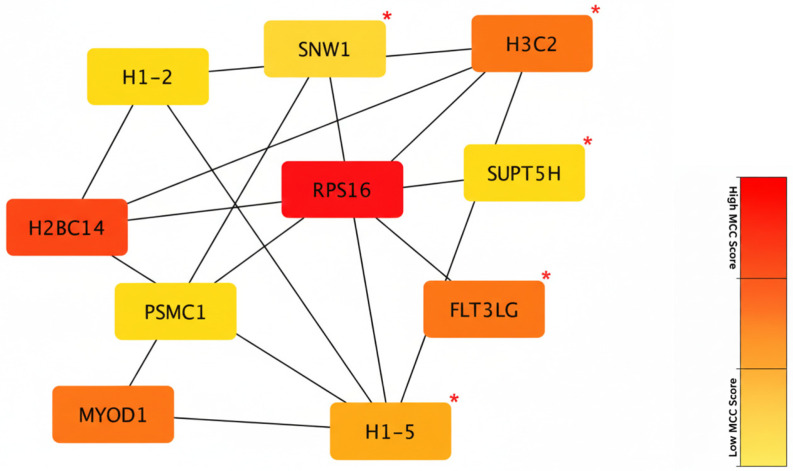
Hub genes according to DEGs. The nodes are ranked based on their Maximal Clique Centrality (MCC) scores, which identify essential nodes in the protein–protein interaction network. The color gradient reflects the correlation with the MCC rank: red indicates the highest-scoring hub genes (top rank), followed by orange (intermediate rank), and yellow (lower rank among top-10) [22]. Red asterisks (*) indicate hub genes with significant survival correlation (*p* < 0.05 for *SNW1*) or a clinical trend (*p* < 0.24 for *H2BC14*, *H3C2*, and *H1-2*).

DEGs between SACs, partial-SACs, and classical CRCs were submitted to the STRING database. In total, 216 nodes and 139 edges were identified. The average node degree was 1.29, and the PPI enrichment score was <0.05 (*p* = 0.0114) (Supplementary Table S3). Then, the top 10 hub genes were identified based on their highest degree of connectivity using the cytoHubba plug-in in Cytoscape. The top ten hub genes were *PSMC1*, *FLT3LG*, *SNW1*, *H3C2*, *H1-2*, *H2BC14*, *H1-5*, *RPS16*, *SUPT5H*, *MYOD1*, *H2BC14*, and *RPS16* genes had the highest scores in terms of interaction (red color); *H3C2*, *FLT3LG*, *MYOD1*, and *H1-5* genes had a medium score (orange color); *PSMC1*, *SNW1*, *H1-2*, and *SUPT5H* genes had the lowest score (yellow color) (Figure 8). *PSMC1*, *SNW1*, *H3C2*, *H1-2*, and *H2BC14* genes had highest expression levels in SACs, while *FLT3LG*, *H1-5*, *RPS16*, *SUPT5H*, and *MYOD1* genes had highest expression levels in partial-SACs. The clinical and prognostic relevance of the identified hub genes was further determined via cBioPortal. Among the top-ranked hub genes, high mRNA expression of *SNW1* was found to be significantly associated with poorer OS (*p* < 0.05). Additionally, elevated expression levels of the *H2BC14*, *H3C2*, and *H1-2* genes demonstrated a notable clinical trend towards reduced DFS (*p* < 0.25).

## 4. Discussion

According to the literature, the prevalence of SACs among CRCs ranges from 5.8% to 12% [5]. In our study, the SAC rate was 9.3%, which is consistent with the range indicated in the literature. The variation in the reported literature is likely due to the application of different histopathological classification systems, inter-observer variability in identifying serrated features, and the diverse geographical origins of the study populations. Despite this relatively stable epidemiological prevalence, the molecular architecture of CRC remains inadequately characterized. Therefore, our in silico study, incorporating cBioPortal data, offers an integrative overview of the distinct gene expression, methylation patterns, and pathway-level changes that collectively distinguish SAC and partial-SAC cases from classical CRCs.

### 4.1. MAPK Pathway and Kinase Activity

Protein kinase, kinase activation and regulation, and serine/threonine protein kinase complex pathways were found to be associated with DEGs among SAC, partial-SAC, and classical CRC groups. According to the literature, the high mutation rate of *KRAS* and *BRAF* in SAC indicates that mitogen-activated protein kinase (MAPK) activation plays an important role in the serrated pathway [24]. Protein kinase activity is controlled by phosphorylating serine, threonine, or tyrosine in specific proteins within cells. Genes in the MAPK pathway also regulate cell activity, such as mitosis, metabolism, and cell death, by phosphorylating serine and threonine [25]. In silico, the relationship between the most enriched genes in SACs and protein kinase complex pathways supports the predominance of MAPK activation in SACs.

In this study, the *KRAS* mutation rate was higher in classical CRCs (classical CRCs: 45.1% vs. SACs: 20%). However, it was detected at a higher rate (50%, *n* = 5/10) in partial-SAC cases. The *KRAS* mutation was detected in 20% (*n* = 4/20) of SACs, which is a lower rate compared to data from the literature. This may be explained by the low number of SAC cases (*n* = 20) evaluated for *KRAS* mutations in this study. *BRAF* mutations were detected in 20% (*n* = 4/20) of SACs and 8.81% *(n* = 17/193) of classical CRCs, indicating a higher prevalence of *BRAF* mutations in SACs. However, since our current in silico study evaluated only 20 cases in SACs for *KRAS* and *BRAF* mutations, our study is not suitable for comparison and further interpretation.

Although no significant enrichment of *KRAS* or *BRAF* mutations was observed in SACs in our dataset, transcriptional and pathway-level signatures provide strong evidence that MAPK activation drives SAC histological variant formation. Consequently, these results imply that functional pathway activation could substitute for mutational status in the characterization of the serrated phenotype. MAPK activation can be achieved through mechanisms other than *KRAS* or *BRAF* mutations, such as epigenetic alterations, overexpression of growth factor receptors, or other upstream signaling molecular mechanisms [26]. This suggests that transcriptional and pathway-level signatures may substitute for mutational status in the characterization of the serrated phenotype.

### 4.2. Cell Morphology

Our research revealed that upregulated and downregulated DEG mRNAs in SACs were associated with cell morphology pathways (such as phagocytic vesicle, microvillus cell components, endocytosis, and non-motile cilium). This may be the reason for the serrated histopathologic appearance. In the literature, it has been emphasized that genes related to cytoskeletal function and cellular components are effective in serrated morphology [27]. Our findings correlate with Mäkinen’s [16] histopathological criteria with regard to underlying molecular changes, indicating that the pathways driving serrated morphology are supported by transcriptional dysregulation.

The *BRAF* V600E mutation and CpG island methylator phenotype (CIMP)-High, known to be associated with SACs, affect not only cell proliferation but also adhesion and polarity, ultimately leading to morphological abnormalities [28]. The high enrichment of pathways related to microvillus cell components and non-motile cilium observed in our analysis suggests a disruption in the apical surface dynamics of the epithelial cells. The non-motile cilium plays a critical role in cell signaling (particularly Hedgehog and Wnt pathways) and cell cycle control, and is regarded as a tumor-suppressor organelle [29].

Abnormalities in genes associated with this structure can lead to an abnormal configuration of the three-dimensional structure and signal transduction of epithelial cells, altering the normal structure of tumor cells and causing the characteristic serrated appearance. In addition, the activity within the phagocytic vesicle and endocytosis pathways represents dynamic processes that control both the cell-to-cell interactions of tumor cells and the exchange of substances with the tumor microenvironment. Dysregulation of these pathways may reflect cytoskeletal remodeling observed during epithelial–mesenchymal transition (EMT) [30]. These molecular events may provide an explanation for the rod-like clusters observed in the invasive fronts of SACs. Our in silico transcriptomic findings are consistent with the literature and demonstrate that the unique morphology in SACs is associated with molecular changes. The alteration of molecular pathways associated with microvilli and cilia underscores the influence of major oncogenic signaling, such as the *BRAF*/CIMP axis, on the disruption of cellular polarity and cytoskeletal architecture.

While the morphological classification in this study relied on manual review by expert pathologists, recent evidence highlights the potential of deep learning (DL) in enhancing CRC diagnosis. For instance, Kather et al. [31] demonstrated that DL algorithms can directly predict MSI and other molecular features from routine H&E-stained digital slides, a task that often challenges human visual assessment. Furthermore, Skrede et al. [32] developed a DL-based prognostic biomarker that outperformed established clinical and pathological parameters in predicting CRC-specific survival. Liu et al. [33] introduced federated learning frameworks that significantly improve the segmentation and classification of serrated polyps across diverse clinical datasets. These developments demonstrate that the unique morphological features of SAC can be quantitatively assessed by developing AI-powered tools, leading to a more reproducible and individualized diagnostic framework in colorectal oncology.

### 4.3. Immunological Pathways

In our study, it was observed that the downregulated mRNA DEGs in SACs were associated with immunological pathways such as PD-L1 expression and PD-1 checkpoint pathway, Th1-2 cell differentiation, T-cell receptor signaling, and Nf-kappa B signaling pathway. In the literature, it has been found that SACs have less immune response and significantly lower peritumoral and intratumoral lymphocytic infiltrate compared to conventional CRCs [34]. Therefore, our findings regarding the downregulation of genes associated with immunological pathways in SACs are consistent with the literature.

The immune response in the serrated pathway plays a critical role in tumor development, growth, and predicting treatment response. SACs are typically characterized by a low level of peritumoral lymphocyte infiltration compared to classical CRCs and MSI-H tumors. SACs show functional enrichment in phagocytosis, B-cell response, and IL-12 inhibition. The increased B-cell response leads to inhibition of anti-tumor immunity, and IL-12 inhibition shifts the immune landscape towards anti-inflammatory behavior. Furthermore, most SACs are MSS, which typically indicates an inadequate response to immune checkpoint therapies [35].

In the literature, an increase in the number of tumor-associated immune cells and higher PD-1/PD-L1 expression have been associated with MSI-H status [36]. Since it is known that SACs are generally MSS, it is expected that tumor-associated immune cell count and PD-L1 expression would be found to be low in SACs. However, this observation needs to be confirmed by new studies comparing PD-L1 expression in SACs with other CRCs.

Immune phenotypes, including immune cells, antigen-presenting mechanisms, and immune checkpoint receptors and inhibitors, are important biomarkers with prognostic and therapeutic implications. Yilmaz et al. [18] found that the expression of immune system markers CD8, FOXP3, and LAG-3 was higher in immune cells in non-SACs compared to SACs. Low CD8 lymphocyte counts are generally associated with decreased expression of immune checkpoint biomarkers, such as PD-L1 and LAG-3. Therefore, consistent with the low CD8 expression levels observed in these cells, lower rates of PD-L1 expression would be expected in SACs. This may explain the association of low-expression DEGs in SACs with PD-L1 expression and the PD-1 checkpoint pathway that we revealed in our in silico study. However, further studies are needed to reach a definitive conclusion on this matter. Additionally, Yilmaz et al. [18] observed that beta-2-microglobulin (*B2MG*) expression in tumor cells was lower in SACs. Since *B2MG* provides tumor neoantigen presentation, the low level of *B2MG* in SACs might also explain the lack of immune response in these tumors [18]. In our study, no in silico data were available regarding *B2MG* expression.

In our study, the downregulation of DEG mRNAs in SAC represents a functional adaptation for immune evasion. By reducing the expression of genes involved in immune signaling, the tumor diminishes its immunogenicity, thereby facilitating survival by circumventing host immune surveillance. Downregulated DEG mRNA in SACs was associated with the immunological pathway, necessitating further investigation of MSI status in these cases. Our study found no statistically significant difference in MSI status between the SAC, partial-SAC, and classical CRC groups (*p* > 0.05 and *q* > 0.05). However, MSS cases were predominant in all groups. The rates of MSI-H cases were similar across all groups (MSI-H rates: SACs 12%; partial-SACs 13.3%; classical CRCs: 15.8%). According to the cBioPortal, there were no significant gene expression differences between the SACs MSS, MSI-L, and MSI-H groups for downregulated mRNA DEGs. Therefore, we believe that MSI status in SAC cases does not directly affect DEGs. This finding strengthens the association between downregulated genes in SACs and immune-related pathways, irrespective of MSI status.

### 4.4. Evaluation of the CIMP Status and Epigenetic Hallmark Signatures

To further characterize the epigenetic landscape of our SAC cohort, we compared the 29 hypermethylated genes identified in SAC and established CIMP-High signatures, specifically the panel proposed by Weisenberger et al. [37]. Although our in silico approach identified a novel prioritized list of targets, significant biological and functional overlaps with classic CIMP markers were observed (Table 1). For instance, the hypermethylation of *PCDHB12*, a member of the clustered protocadherins, aligns with the known epigenetic silencing patterns of CIMP-High CRCs [38]. Additionally, the methylation of *MAP3K7CL* supports the critical role of MAPK pathway dysregulation in the serrated carcinogenic route [39]. These findings demonstrate that our SAC-associated methylation profile is consistent with established CIMP hallmarks while identifying novel subtype-specific markers, like *ZNF80* and *TTN*.

### 4.5. Hub Genes

Hub genes, identified in PPI network analyses, are the genes with the highest degree of connectivity in a cellular network and often play a central role in regulating biological processes. These genes are key components of critical molecular mechanisms, including signal transduction, cell cycle regulation, and apoptosis. Altered expression or mutation of hub genes can contribute to tumor development and progression [41,42,43]. Numerous in silico analyses in CRCs have revealed strong associations between hub genes and the tumor microenvironment, immune response, and metastatic potential. Therefore, identifying hub genes enhances the understanding of the structural properties of the biological network and facilitates the identification of potential diagnostic biomarkers and therapeutic targets [44,45].

Our in silico analysis revealed that all of these genes exhibited enrichment in either the SAC (*H2BC14*, *H3C2*, *PSMC1*, *SNW1*, and *H1-2* genes) or partial-SAC groups (*RPS16*, *FLT3LG*, *MYOD1*, *H1*-5, and *SUPT5H* genes). These genes regulate chromatin structure (*H1-2*, *H1-5*, *H3C2*, and *H2BC14*), proteasomal activity and apoptosis (*PSMC1*), WNT/MAPK signaling pathway (*SNW1*), immune cell recruitment and immunotherapy response (*FLT3LG*), transcriptional elongation and oncogenic signaling (*SUPT5H*), TGF-β1 and Smad signaling pathways (*MYOD1*), and cell proliferation and metastasis (*RPS16*) [46,47,48,49,50,51,52,53,54]. Considering that mutations in *the PSMC1* and *SNW1* genes inhibit apoptosis, it is expected that these genes are enriched in SACs. Because it is known that one of the mechanisms responsible for serration is apoptosis inhibition. The presence of chromatin-related center genes supports the idea that epigenetic dysregulation is of critical importance for the serrated pathway. This finding correlates with the CIMP-High model [2].

Furthermore, the co-occurrence of immune-related hub genes (*FLT3LG*, *H3C2*) with immunosuppressive pathway signatures supports that SACs may harbor selective immune-regulatory alterations. This dichotomy at the hub gene level has not been elucidated in previous studies. While *FLT3LG* and *H3C2* are typically associated with favorable immunotherapy outcomes in various cancers, their enrichment in our serrated subgroups (partial-SACs and SACs, respectively) presents a paradoxical finding given the overall downregulation of PD-1/PD-L1 and NF-κB pathways in these cases. We propose that in the unique molecular landscape of SAC, the predictive value of these markers may be neutralized by the concurrent epigenetic silencing of other critical immune-recruitment factors [55].

It should be noted that detailed immunotherapy treatment records and clinical response data were not available for TCGA cases, which limits our ability to directly correlate molecular signatures with therapeutic outcomes. Despite this, our in silico analysis revealed a paradoxical enrichment of *FLT3LG* in serrated subgroups, which presents a complex clinical picture regarding the immune microenvironment. Previous studies have demonstrated that *FLT3LG* expression positively correlates with the infiltration of cytotoxic T cells, activated dendritic cells, NK cells, and Th1 cells, acting as a potential prognostic marker that enhances immunotherapy efficacy in various cancers [49]. Specifically, *FLT3LG* has been shown to modulate immune cell infiltration and improve the response to anti-PD-1 therapy in CRCs [56]. However, the ‘immune-cold’ phenotype we observed in SACs—characterized by the downregulation of PD-L1 and T-cell receptor signaling pathways—suggests that this potential immune activation is being suppressed.

Rather than indicating a responsive immune response-rich tumor, the enrichment of *FLT3LG* might represent a compensatory but ineffective immune activation attempt within an otherwise immune-cold microenvironment [57]. Consequently, we hypothesize that *FLT3LG* should be investigated as a context-specific biomarker for immunotherapy resistance in serrated malignancies, suggesting that these patients may require alternative strategies, such as combined epigenetic modulators or kinase inhibitors, to overcome immune evasion [58]. As suggested by Dang et al. [55], such immunotherapy resistance in CRC can potentially be overcome by combining immune checkpoint inhibitors with epigenetic modulators. These hub genes, which constitute the center of DEGs among SAC and non-serrated CRC groups, are potential therapeutic targets. Therefore, further studies on these hub genes in SACs are needed.

The molecular landscape and the hub genes identified in this study, such as *SNW1*, *FLT3LG*, and the H2B histone family, provide a high-priority roadmap for future translational research. While the current study is focused on a comprehensive in silico discovery phase, these findings serve as a foundational baseline for subsequent wet-lab validation. Following the methodology of Zhang et al. [59], transitioning from computational identification to experimental validation is essential to confirm these targets as reliable biomarkers. Specifically, we propose that future research focus on validating the protein expression of hub genes via immunohistochemistry across SAC and classical CRC cohorts to assess their diagnostic utility. Furthermore, to elucidate the functional mechanisms behind the observed serrated morphology and apoptosis inhibition, in vitro CRISPR-Cas9 knockdown assays of identified chromatin-related genes, such as *H3C2* and *H2BC14*, should be conducted in SAC-specific cell lines. By defining these specific targets, our work narrows the gap between high-throughput genomic data and targeted functional research in this rare colorectal cancer subtype.

The molecular landscape of SAC is increasingly recognized for its complex interplay with host metabolic factors [60]. Although systemic parameters are not directly available in public genomic databases like TCGA, our findings can provide a potential genetic basis for these clinical observations. Metabolic dysregulation, often manifested as insulin resistance (high triglyceride–glucose index), has been linked to the activation of the PI3K/AKT/mTOR signaling pathway, which is frequently altered in CRCs [60,61,62]. By characterizing these epigenetic and mutational signatures, our study offers a baseline for future clinical studies to investigate how specific metabolic thresholds correlate with the genomic changes in SAC patients.

### 4.6. Survival

Despite the distinct morphological features and molecular background associated with SACs, our survival analyses did not demonstrate a significant difference in either DFS or OS between SAC, partial-SAC, and classical CRC groups. While the median DFS and OS values were generally shorter in serrated subtypes, this did not reach statistical significance, likely due to the limited number of SAC and partial-SAC cases. A review of previous studies reveals conflicting findings regarding survival outcomes in SACs. Some studies have indicated a more aggressive clinical course associated with CIMP, *BRAF* mutation, and MSI [2,63]. However, another study observed results similar to those in conventional CRC [18]. Our findings support the idea that the prognosis of SAC may vary, depending not only on morphology but also on underlying molecular alterations. Further research is necessary to improve the accuracy of predicting the clinical behavior of these tumors. In our study, we observed that clinical staging parameters—including T, N, and M stages, as well as overall pathological stages—were remarkably consistent across the SAC, partial-SAC, and classical CRC groups (*p* > 0.05). This clinical homogeneity suggests that the molecular divergence we identified is driven by intrinsic biological differences rather than disparities in disease progression at the time of diagnosis. Furthermore, the lack of a statistically significant difference in survival between these groups may be attributed to the balanced distribution of clinical stages. Since stage is one of the most powerful predictors of prognosis in colorectal cancer, the similar staging profiles likely masked the potential impact of SAC-specific molecular signatures on survival outcomes within this particular dataset. Nevertheless, the distinct ‘molecular silencing’ and immune evasion patterns observed in SAC provide a compelling rationale for further investigation into how these biological traits might influence treatment response, independent of the clinical stage.

The clinical relevance of our hub genes was further demonstrated by their significant impact on patient survival in the TCGA cohort. Specifically, elevated *SNW1* expression was significantly correlated with reduced OS (*p* < 0.05), while chromatin-related genes *H2BC14*, *H3C2*, and *H1-2* showed a notable trend towards poorer DFS (*p* < 0.25). These findings suggest that beyond their structural roles, these genes serve as potent prognostic biomarkers in SAC. Given these links to patient outcomes, these hub genes should be prioritized for future experimental validation as potential therapeutic targets.

Our integrated framework for SAC risk stratification provides a significant advantage over CRC models, such as the T, N, M staging system, which primarily relies on the histopathological examination. In contrast, our approach incorporates a multi-dimensional perspective by integrating molecular pathways and epigenetic hallmarks. By identifying high-priority hub genes, our model offers molecular insights into the unique pathophysiology of the serrated pathway, an area often underrepresented in generalized risk-scoring systems. This integrated molecular perspective facilitates a more personalized assessment of SAC patients, potentially identifying high-risk individuals who may be classified as low-risk under traditional staging criteria due to their distinct biological characteristics.

In addition to molecular and morphological markers, the clinical management of CRC patients involves biochemical indicators that reflect the patient’s physiological state during surgical interventions. A notable example is butyrylcholinesterase (BChE), an enzyme synthesized by the liver that has recently been recognized as a significant prognostic marker in colorectal cancer operations. Low serum levels of BChE at the time of diagnosis or surgery are often associated with systemic inflammation, malnutrition, and advanced tumor stage [64,65]. Recent clinical studies, such as those by Alburiahi et al. [64] and Santarpia et al. [65], have demonstrated that diminished BChE activity serves as an independent predictor of poor overall survival and increased postoperative complications in CRC patients. This enzyme reflects the cancer-related systemic inflammatory response, and its integration into the clinical assessment alongside our identified molecular signatures—such as the downregulated immunological pathways in SACs—could provide a more comprehensive overview of the patient’s recovery potential and long-term prognosis.

Our findings outline a mechanistic model in which SAC development is characterized by the MAPK pathway, chromatin-mediated transcriptional reprogramming, immune pathway suppression independent of MSI status, and cytoskeletal and epithelial remodeling underlying serrated morphology. The combination of these molecular features indicates that SAC is not just a histological variant of CRC. Instead, it is a biologically separate entity that is caused by combined somatic, epigenetic, and transcriptional changes.

Our current study detected significant genetic and epigenetic changes in CRC cases with and without serrated features. However, our results need to be further supported by in vitro and in vivo methods. Although common molecular changes have been detected in the development of SAC and conventional CRCs, studies have identified differences in a limited number of genes. The lack of a specific molecular signature for the serrated pathway suggests that further research is needed to elucidate the relationship between the serrated cancer pathway and SAC.

## 5. Conclusions

In this study, we investigated the potential molecular differences in the TCGA dataset that could be linked to the serrated pathway in CRCs. Although several candidate genes and pathways were identified, their clinical relevance necessitates validation through functional assays and larger patient cohorts. Our integrated multi-omics analysis identifies *PSMC1*, *SNW1*, and histone-related genes *H3C2*, *H1-2*, and *H2BC14* as the most promising molecular targets for SAC. These genes are hub genes showing enrichment in SACs and providing a critical foundation for future precision therapies. Further studies are needed to investigate the potential of kinase inhibition or immune checkpoint modulation as targeted therapy and of epigenetic-targeted treatment strategies in this colorectal cancer subtype.

### Limitations

The cases were evaluated based on the digital slides uploaded to TCGA database, and it is possible that not all areas of the tumor were uploaded to this database. Only the uploaded digital slides were evaluated. While the histopathological classification was confirmed by two independent pathologists through a consensus approach, inter-observer agreement was not formally quantified using Cohen’s kappa coefficient. The cBioPortal does not include MSI data for all cases.

A notable limitation of the present study is the reliance on in silico bioinformatic analyses without direct experimental validation or functional in vitro assays. Therefore, our findings should be interpreted as a robust prioritized list of molecular targets that require further validation in controlled laboratory environments to definitively elucidate their role in SAC pathogenesis.

A significant limitation of this study is the inherent sample size imbalance between classical CRCs (*n* = 546) and SACs (*n* = 59). To address this, a G* power (version 3.1) analysis was conducted to evaluate the reliability of our survival findings. Given the current sample distribution, the statistical power to detect subtle survival differences may be limited, potentially explaining the lack of statistical significance (*p* > 0.05) despite observed differences in survival times. Future studies with more balanced cohorts are necessary to confirm if these molecular signatures translate into distinct clinical outcomes.

## Figures and Tables

**Table 1:** Comparison of SAC-associated hypermethylated genes with established CIMP signatures.

Comparison Criteria	Established CIMP Markers [37]	SAC-Associated Hypermethylated Genes (Present Study)	Clinical/Biological Significance
Prominent Genes	*MLH1*, *CDKN2A*, *CACNA1G*, *NEUROG1*, *RUNX3*, *SOCS1*, *IGF2*, and *CRABP1*	ZNF80, TTN, OR2AG2, DGCR5, MAP3K7CL, and PCDHB12	Both groups reflect the epigenetic silencing process, which is the primary mechanism of carcinogenesis in the serrated pathway
Pathway Association	DNA repair (*MLH1*), Cell cycle (*CDKN2A*)	MAPK signaling (MAP3K7CL) [39], chromatin regulation (DGCR5) [40], and cell adhesion (*PCDHB12*) [38]	Directly associated with *MAPK/BRAF* activation and morphological changes (serration) characteristic of SAC
CIMP-High Alignment	Standard markers defining the CIMP-High phenotype	Directly overlaps with the PCDH cluster (e.g., *PCDHB12*) found in established CIMP-High panels	Confirms that SAC cases harbor a distinct epigenetic signature that is deeper and more subtype-specific than the standard CIMP-High profile

## Data Availability

The data presented in this study are available in the cBioPortal for Cancer Genomics (cBioPortal) website (https://www.cbioportal.org/study/summary?id=coadread_tcga (accessed on 11 January 2026)). TCGA numbers of cases showing serrated features had been provided in Supplementary Table S1, and can be obtained in TCGA—National Cancer Institute (NCI) (https://www.cancer.gov/ccg/research/genome-sequencing/tcga (accessed on 11 January 2026)).

## Supplementary Materials

The following supporting information can be downloaded at: https://www.mdpi.com/article/10.3390/cimb48020179/s1.

## Abbreviations

AJCC	American Joint Committee on Cancer
B2MG	Beta-2-microglobulin
BP	Biological process
BChE	Butyrylcholinesterase
cBioPortal	cBioPortal for Cancer Genomics
CC	Cellular component
CIMP	CpG island methylator phenotype
CRC	Colorectal carcinoma
DEGs	Differentially expressed genes
DFS	Disease-free survival
DL	Deep learning
FDR	False discovery rate
GO	Gene Ontology
KEGG	Kyoto Encyclopedia of Genes and Genomes
MAPK	Mitogen-activated protein kinase
MCC	Maximal clique centrality
MF	Molecular function
MSS	Microsatellite stable
MSI	Microsatellite instability
MSI-H	Microsatellite instability-High
MSI-L	Microsatellite instability-Low
NCI	National Cancer Institute
ORA	Over-representation analysis
OS	Overall survival
Partial-SAC	CRC with partial SAC morphology
PPI	Protein–protein interaction
SAC	Serrated adenocarcinoma
SSL	Sessile serrated lesion
STRING	The Search Tool for the Retrieval of Interacting Genes/Proteins
TCGA	The Cancer Genome Atlas Program
TSA	Traditional serrated adenoma
WebGestalt	WEB-based Gene SeT Analysis Toolkit
WHO	World Health Organization
