# Supplementary material for: Genetic and Epigenetic Mechanisms in Serrated Adenocarcinomas and Classical Colorectal Carcinomas: An In Silico Study
Source: Curr Issues Mol Biol. 2026 Feb 4;48(2):179. doi: 10.3390/cimb48020179 (PMC12939040; doi:10.3390/cimb48020179)

The pathway analysis for differentially expressed hypermethylated genes in SACs

Gene Ontology: Biological Process

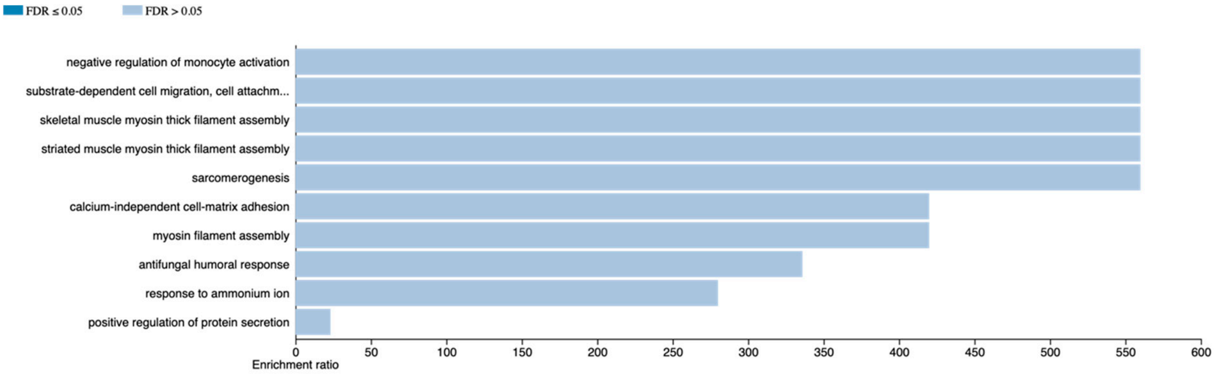

Gene Ontology: Molecular Function

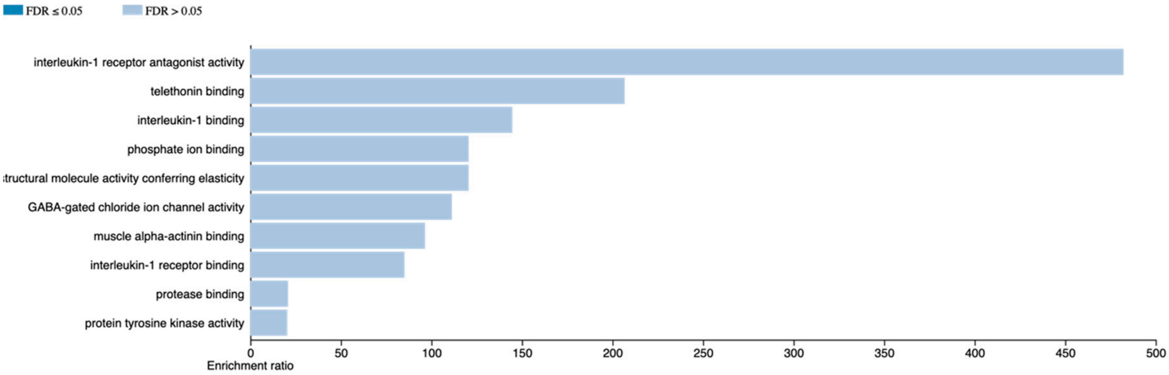

Gene Ontology: Cellular Component

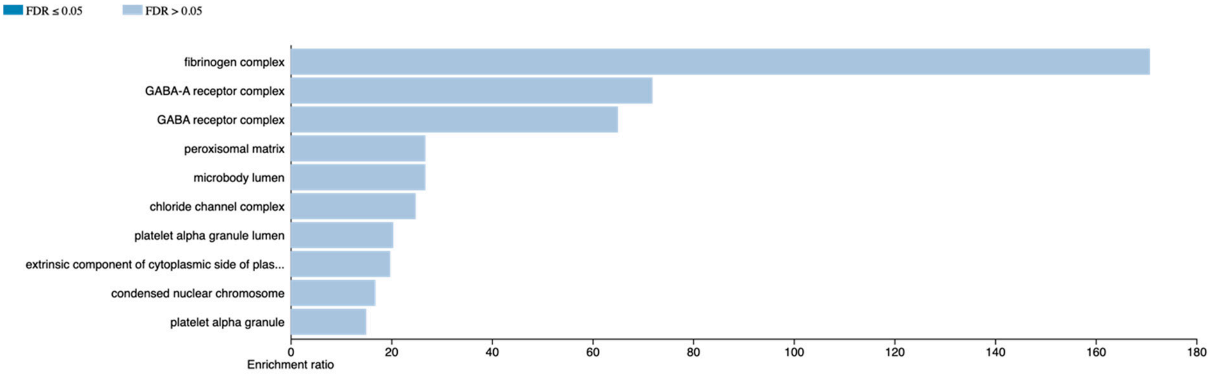

KEGG pathway

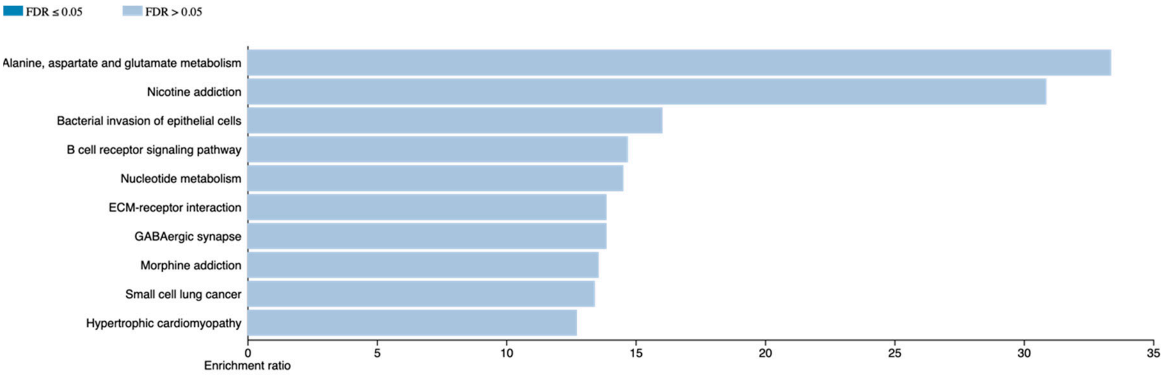

Supplement: Supplementary file 1 [file cimb-48-00179-s001.zip › Supplementary Figure S2.pdf]
