# Supplementary material for: Genetic and Epigenetic Mechanisms in Serrated Adenocarcinomas and Classical Colorectal Carcinomas: An In Silico Study
Source: Curr Issues Mol Biol. 2026 Feb 4;48(2):179. doi: 10.3390/cimb48020179 (PMC12939040; doi:10.3390/cimb48020179)
Supplement: Supplementary file 1 [file cimb-48-00179-s001.zip › Supplementary Table S1.pdf]

| Serrated Adenocarcinoma | Partial-Serrated Adenocarcinoma |
|---|---|
| TCGA-A6-3807 | TCGA-A6-2685 |
| TCGA-A6-5657 | TCGA-A6-5656 |
| TCGA-A6-5659 | TCGA-AA-3548 |
| TCGA-A6-5660 | TCGA-AA-A01C |
| TCGA-A6-5661 | TCGA-G4-6627 |
| TCGA-A6-5665 | TCGA-CM-6676 |
| TCGA-A6-6650 | TCGA-F4-6807 |
| TCGA-AA-3495 | TCGA-DM-A28M |
| TCGA-AA-3525 | TCGA-QG-A5YX |
| TCGA-AA-3526 | TCGA-AA-A02E |
| TCGA-AA-3660 | TCGA-AZ-4684 |
| TCGA-AA-3664 | TCGA-DY-A1DG |
| TCGA-AA-3688 | TCGA-A6-2672 |
| TCGA-AA-3712 | TCGA-A6-2677 |
| TCGA-AA-3812 | TCGA-A6-2683 |
| TCGA-AA-3831 | TCGA-A6-6137 |
| TCGA-AA-3842 | TCGA-A6-A56B |
| TCGA-AA-3846 | TCGA-AA-3522 |
| TCGA-AA-3939 | TCGA-AA-3841 |
| TCGA-NH-A8F7 | TCGA-AA-3870 |
| TCGA-AY-4071 | TCGA-AA-3973 |
| TCGA-AA-A01V | TCGA-AA-A00E |
| TCGA-AA-A02O | TCGA-AA-A03J |
| TCGA-AU-6004 | TCGA-F4-6570 |
| TCGA-CM-5344 | TCGA-AZ-6601 |
| TCGA-CM-5348 | TCGA-AG-3612 |
| TCGA-D5-6535 | TCGA-AF-6136 |
| TCGA-D5-6539 | |
| TCGA-G4-6321 | |
| TCGA-AZ-6599 | |
| TCGA-F4-6459 | |
| TCGA-F4-6569 | |
| TCGA-G4-6588 | |
| TCGA-AZ-6606 | |
| TCGA-CA-5254 | |
| TCGA-CA-5255 | |
| TCGA-CA-6716 | |
| TCGA-G4-6309 | |
| TCGA-AZ-4315 | |
| TCGA-AZ-4682 | |
| TCGA-CM-5341 | |
| TCGA-D5-5539 | |
| TCGA-F5-6571 | |
| TCGA-F5-6863 | |
| TCGA-AG-3890 | |
| TCGA-DC-6681 | |
| TCGA-G5-6235 | |
| TCGA-AG-A01L | |
| TCGA-AG-3731 | |
| TCGA-AF-3913 | |
| TCGA-AF-5654 | |
| TCGA-AG-3602 | |
| TCGA-AA-3516 | |
| TCGA-AA-A00J | |
| TCGA-AA-3956 | |
| TCGA-CA-6719 | |
| TCGA-AD-6888 | |
| TCGA-D5-6923 | |
| TCGA-CM-4750 | |
